# Ocular Neurosyphilis in an Immunocompetent Patient Without HIV: A Diagnostic Challenge

**DOI:** 10.7759/cureus.95895

**Published:** 2025-11-01

**Authors:** Amir Fanaei, Adam J Metivier, Peter Morreale

**Affiliations:** 1 Internal Medicine, Saint Louis University School of Medicine, Saint Louis, USA

**Keywords:** csf-vdrl, hiv-negative, ocular neurosyphilis, panuveitis, vision loss

## Abstract

Ocular syphilis is a rare but significant manifestation of *Treponema pallidum* infection that may present at any stage of the disease. It can affect virtually any ocular structure, with panuveitis being among the most frequent findings. This case report describes a 44-year-old HIV-negative man who presented with progressive unilateral vision loss and was diagnosed with ocular neurosyphilis despite a negative cerebrospinal fluid (CSF) Venereal Disease Research Laboratory (VDRL) test. His diagnosis was supported by positive serum and CSF treponemal antibodies, clinical findings, and treatment response. This case emphasizes the importance of maintaining a high index of suspicion for neurosyphilis even in immunocompetent patients and highlights the diagnostic challenges associated with this condition.

## Introduction

Syphilis is a chronic systemic infection caused by the spirochete *Treponema* [1-3]. Its clinical manifestations vary widely and progress through four stages: primary, secondary, latent, and tertiary [1,2]. Although neurosyphilis is classically associated with late-stage disease, it can occur at any time after initial infection [2,4]. Ocular syphilis, a form of neurosyphilis, is rare and potentially vision-threatening [1-5]. It is most commonly seen in patients co-infected with HIV [6]. However, it can also occur in immunocompetent individuals, making timely recognition critical [6-9]. The disease can mimic a wide range of infectious and inflammatory conditions, and diagnosis may be missed or delayed if not actively considered [1,3,5].

Ocular manifestations of syphilis include anterior uveitis, posterior uveitis, panuveitis, optic neuritis, retinitis, and chorioretinitis [1,3,9]. Panuveitis is among the more common ocular findings in neurosyphilis, but it remains rare overall [1,3,5,6,10]. The diagnosis is typically supported by serologic testing, cerebrospinal fluid (CSF) analysis, and neuro-ophthalmic imaging, though standard tests such as CSF-VDRL (Venereal Disease Research Laboratory) may yield false-negative results [11-14]. This report presents a case of ocular neurosyphilis in an HIV-negative patient with a negative CSF-VDRL but positive treponemal antibodies and an excellent response to treatment.

## Case presentation

A 44-year-old man with a history of hypertension and gastroesophageal reflux disease presented with progressive vision loss in his left eye over the course of three weeks. He initially reported mild blurriness and pressure, which worsened to complete vision loss. He was evaluated by ophthalmology and diagnosed with panuveitis. Initial ophthalmoscopy indicated a normal right eye disc, cup-to-disc ratio, macula, blood vessels, and peripheral retina. However, the left eye was limited by poor visualization, likely secondary to intraocular inflammation. On slit-lamp examination, the left eye demonstrated greater than two anterior chamber cells and flare, with keratic precipitates noted inferiorly. The fundus view was significantly limited due to vitritis and media haze, precluding detailed assessment of the disc, macula, and vasculature. No hemorrhages or cotton wool spots were visualized in the limited view. Follow-up examinations documented gradual clearing of anterior chamber inflammation but persistent difficulty with posterior visualization. The patient declined formal ophthalmoscopy after hospital discharge. He was prescribed topical atropine and prednisolone and received a local steroid injection.

Baseline ophthalmologic findings revealed a visual acuity of 20/20 in the right eye and 20/400 in the left eye without pinhole improvement. Tonometry showed intraocular pressures of 10 mmHg bilaterally. The right pupil was round, briskly reactive to light, and without afferent pupillary defect, while the left pupil was nonreactive with a relative afferent pupillary defect (positive by reverse testing). Extraocular movements and visual fields were full bilaterally. Both eyes were pharmacologically dilated with 1.0% mydriacyl and 2.5% phenylephrine for further evaluation.

**Figure 1:**
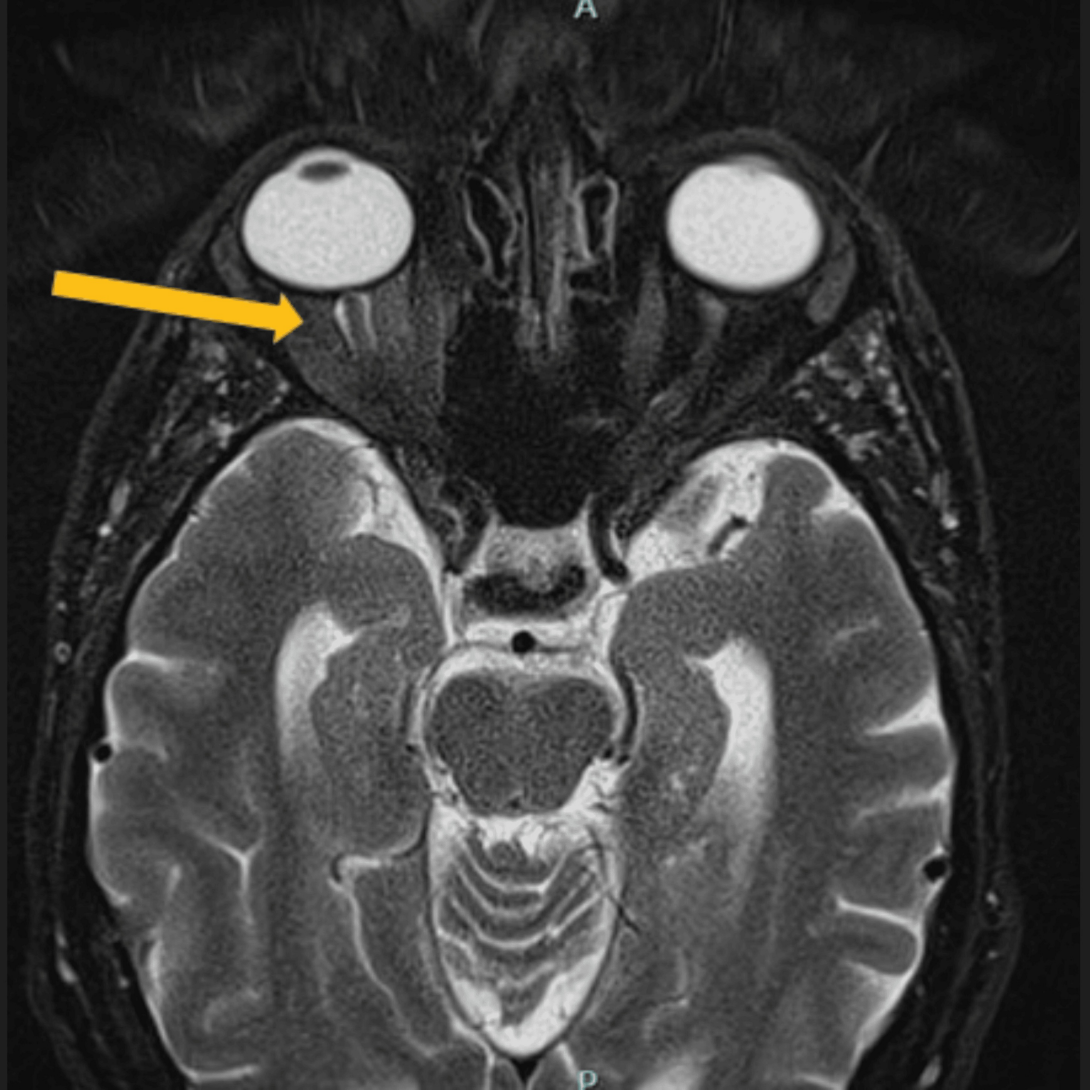
An MRI Scan of the Brain and Orbit Showing a T2 Axial View MRI of the orbits showing mild enhancement of the right optic nerve sheath (orange arrow), consistent with perineuritis. The patient's symptomatic vision loss was in the left eye, illustrating subclinical or asymmetric optic nerve involvement in ocular neurosyphilis.

Given concern for an infectious etiology, laboratory testing was ordered, including serum rapid plasma reagin (RPR), treponemal-specific antibodies, and a full sexually transmitted infection (STI) panel. A lumbar puncture was performed for CSF analysis, including VDRL and tests for viral, fungal, and mycobacterial pathogens. Ophthalmology, Neurology, and Infectious Disease specialists were consulted. An MRI of the brain and orbits was obtained, showing right eye optic neuritis (Figure 1).

Further evaluation was complicated by the incidental discovery of cavitary lung lesions, prompting additional workup, including serial sputum samples and QuantiFERON testing, which were negative for tuberculosis.

Despite a negative CSF-VDRL, the patient had a reactive RPR (1:32 titer), and both serum and CSF were positive for *Treponema pallidum* antibodies. Given the ocular findings, clinical course, and positive serologies, a diagnosis of ocular neurosyphilis was made (Table 1).

**Table 1:** Comprehensive CSF and Laboratory Findings Supporting Neurosyphilis CSF: cerebrospinal fluid, PCR: polymerase chain reaction, WBC: white blood count, VDRL: Venereal Disease Research Laboratory, FTA-ABS: fluorescent treponemal antibody absorption, RPR: rapid plasma reagin, AFB: acid-fast bacteria

Test Type	Test	Result	Normal Range
CSF analysis	WBC	8 cells/μL (85% lymphocytes)	0–5 cells/µL (40–80% lymphocytes)
	Protein	36 mg/dL	15–60 mg/dL
	Glucose	65 mg/dL	45–80 mg/dL
	VDRL	Non-reactive	Negative (<1:8)
	FTA-ABS	Reactive	Negative
	PCR HSV, VZV	Negative	Negative
Infectious workup	RPR	Reactive (1:32 titer)	Negative (<1:8)
	*Treponema pallidum* antibodies	Reactive	Negative
	HIV	Negative	Negative
	AFB smear	Negative	Negative
	Mycobacterial culture	Negative	Negative

He was treated with intravenous penicillin G (three million units every four hours) for 14 days per CDC guidelines. The patient experienced complete visual recovery following treatment. No additional antimicrobial therapy was needed, and he was discharged to a skilled nursing facility to complete his antibiotic course. He continues to follow up as an outpatient with ophthalmology and remains symptom-free.

## Discussion

This case illustrates a classic but diagnostically challenging presentation of ocular neurosyphilis in an HIV-negative patient. While neurosyphilis is more frequently seen in individuals with HIV, its occurrence in immunocompetent patients highlights the need for clinical vigilance regardless of immune status [4,7,15].

Panuveitis, although one of the more common ocular manifestations of neurosyphilis, is also associated with a number of autoimmune and granulomatous conditions such as sarcoidosis and Behçet's disease [12]. These are often treated with corticosteroids, which could potentially worsen syphilitic infection if misapplied [5,12]. In this patient, an initial steroid injection was administered before a definitive infectious diagnosis, further underscoring the importance of prompt and accurate identification of syphilitic uveitis (Table 2) [4,15,16].

**Table 2:** Key Features Differentiating Panuveitis and Neurosyphilis TB: tuberculosis, ANA: antinuclear antibody, ACE: angiotensin-converting enzyme, RPR: rapid plasma reagin, VDRL: Venereal Disease Research Laboratory, FTA-ABS: Fluorescent Treponemal Antibody Absorption, IV: intravenous Table created by the authors based on [4,10]

Feature	Panuveitis [10]	Neurosyphilis [4]
Cause	Autoimmune (e.g., sarcoidosis, Behçet’s), infections (e.g., TB, HSV, toxoplasmosis)	Syphilis infection spreading to the CSF
Symptoms	Eye pain, redness, photophobia, floaters, and blurred vision	Similar to panuveitis
Diagnosis	Clinical exam and targeted labs (ANA, ACE, QuantiFERON, etc.), chest X-ray, biopsy	Blood tests for syphilis (RPR, VDRL) and spinal fluid testing for FTA-ABS
Treatment	Corticosteroids, immunosuppressants, antimicrobial agents	IV penicillin G for 10–14 days and steroids for severe inflammation

Diagnostic confirmation of neurosyphilis can be difficult, with up to 40% of patients having completely normal CSF results, particularly in the setting of a negative CSF-VDRL, which lacks sensitivity [15]. Studies show the sensitivity of CSF-VDRL ranges from 0 to 50%, with many studies reporting a sensitivity of ≤30% [3,11,14,17]. The presence of treponemal antibodies in CSF, coupled with consistent clinical findings and a positive therapeutic response to penicillin, is often sufficient to establish the diagnosis [2,11,14,18].

Previous reports have shown that ocular syphilis most often occurs in immunocompromised patients, particularly those with HIV [6,19]. Other cases in the literature highlight the variability in neurosyphilis presentation, with some patients presenting initially with ocular symptoms, others with atypical otic involvement, and many in the setting of HIV co-infection [7,8,19,20]. In a large series by Moradi et al., over 50% of patients with ocular syphilis were HIV-positive, and visual recovery was often incomplete despite appropriate therapy [6]. Similarly, other reports of HIV-negative patients frequently describe only partial recovery of vision [7,9,12]. By contrast, our patient was immunocompetent and achieved complete visual recovery following a 14-day course of intravenous penicillin, consistent with CDC guidelines. This highlights the importance of early recognition and treatment, even in patients without traditional risk factors [2].

Ophthalmoscopic descriptions in prior literature frequently report optic disc edema, retinal vasculitis, and chorioretinitis [3,6,9,12]. Our case was limited by poor posterior visualization secondary to vitritis, which restricted assessment of the optic disc and macula. Follow-up ophthalmoscopy and MRI were not obtained due to patient refusal, representing a limitation of this report. Nonetheless, an MRI demonstrating optic neuritis at presentation, together with serologic and CSF findings, was sufficient to establish the diagnosis [12]. The pathophysiology of optic neuritis in neurosyphilis is multifactorial. *Treponema pallidum* can invade the optic nerve either hematogenously or through direct meningeal extension, leading to perineuritis or true optic neuritis. Small-vessel endarteritis causes ischemic demyelination, and immune-mediated inflammation may exacerbate axonal injury. These mechanisms explain both the optic nerve sheath enhancement seen on MRI and the rapid visual recovery following antibiotic therapy [3,5,6,12,15].

This comparison reinforces the novelty of our case: an immunocompetent, HIV-negative patient with negative CSF-VDRL who achieved full visual recovery [11,18]. Finally, this case underscores the limitations of relying solely on CSF-VDRL for diagnosis, demonstrates that immunocompetent patients can develop severe ocular disease, and highlights the importance of early initiation of guideline-based therapy [2,4,11,14]. Although follow-up imaging was not obtained, the patient's clinical course and full recovery provide strong support for the diagnosis of ocular neurosyphilis.

## Conclusions

Ocular neurosyphilis is a rare but serious complication of syphilis that requires early recognition and treatment to prevent permanent vision loss. Our case is notable for its occurrence in an immunocompetent, HIV-negative patient who achieved complete visual recovery, an uncommon outcome in published reports. Clinicians should remain aware of the limitations of CSF-VDRL testing and rely on a combination of clinical, serologic, and radiologic findings to guide diagnosis. While our report is limited by the absence of follow-up ophthalmoscopy and MRI, the patient's excellent response to timely intravenous penicillin underscores the importance of early initiation of guideline-based therapy in suspected cases.
